# Medication persistence over 2 years of follow-up in a cohort of early rheumatoid arthritis patients: associated factors and relationship with disease activity and with disability

**DOI:** 10.1186/ar2620

**Published:** 2009-02-19

**Authors:** Virginia Pascual-Ramos, Irazú Contreras-Yáñez, Antonio R Villa, Javier Cabiedes, Marina Rull-Gabayet

**Affiliations:** 1 Department of Immunology and Rheumatology, Instituto Nacional de Ciencias Médicas y Nutrición Salvador Zubirán, Vasco de Quiroga 15-Colonia Sección XVI-Tlalpan, México City, 14000, México; 2 Clinic Epidemiology Unit, Instituto Nacional de Ciencias Médicas y Nutrición Salvador Zubirán, Vasco de Quiroga 15-Colonia Sección XVI-Tlalpan, México City, 14000, México

## Abstract

**Introduction:**

Aggressive treatment with disease-modifying antirheumatic drugs (DMARDs) plays a major role in improving early rheumatoid arthritis (RA) patient outcomes. Persistence and adherence with medication occurs variably (20% to 70%). The objectives of the study were to determine medication persistence (MP) in early RA patients over 13 consecutive visits each 2 months apart, to investigate the relationship between MP and disease activity, disability and structural damage, and to identify baseline prognosticators.

**Methods:**

Charts from 75 patients of an early RA cohort were reviewed. At each visit, a rheumatologist interviewed patients regarding therapy, scored disease activity with the 28-joint disease activity score (DAS28) and disability with the health assessment questionnaire (HAQ), and recorded comorbidities and treatment. A complete medical history was obtained at baseline. MP was defined as the duration of time from initiation to discontinuation of at least one DMARD and/or corticosteroids for at least 1 week and was reported as a dichotomous variable at consecutive evaluations. Structural damage was defined by detection of new erosions on radiography. Descriptive statistics, Student's *t *test, the chi-squared test, and logistic regression analyses were used.

**Results:**

The proportion of MP patients decreased from 98% at 2 months to 34% at 2 years. MP patients (n = 32) had similar DAS28 to non-MP patients (n = 53) at initial visits, lower DAS28 and greater DAS28 improvements at follow-ups (*P *≤ 0.05 at visits 4, 6, 7 and 9) and reached sustained remission (≥ 3 consecutive visits with DAS28 < 2.6) more frequently (82.8% versus 46.5%, *P *= 0.003) and earlier (7.7 ± 4.6 versus 13.6 ± 5.7 months, *P *= 0.001) than non-MP patients. MP patients had similar baseline HAQ scores, but lower HAQ scores at follow-up (*P *≤ 0.05 at visits 3, 5, 6, 7, 9, 10 and 13). More non-MP patients developed erosive disease than MP patients (26.8% versus 17.9%, *P *= 0.56). Older age at baseline was associated with therapy discontinuation (odds ratio = 1.1, 95% confidence interval = 1.007 to 1.103, *P *= 0.02).

**Conclusions:**

Discontinuation of DMARDs was frequent and progressive in an early RA cohort. Patients with persistence on therapy were younger, had lower disease activity and disability during follow-up, and reached sustained remission more frequently and earlier than patients without it. MP should intentionally be evaluated during follow-up of early RA patients, as it seems to play a major role in outcome.

## Introduction

Rheumatoid arthritis (RA) is a chronic inflammatory disease that may result in significant disability, morbidity and increased mortality [1]. In recent years, earlier aggressive treatment with disease-modifying drugs (DMARDs) has been shown to play a major role in improving patient outcomes. Those benefits will be achieved only if patients follow prescribed treatment regimens reasonably closely.

Both adherence and compliance are words commonly used to describe how patients take their medication. Traditionally, adherence to therapy has been defined as 'the number of patients continuing treatment with a particular drug prescribed by their health care provider, regardless of the clinical response' [2,3]. Guidance regarding the meaning of compliance (adherence) and persistence has been proposed recently [4]. Accordingly, *medication compliance *(synonym *adherence*) is defined as 'the extent to which a patient acts according to the prescribe interval and dose of a dosing regimen' and *medication persistence *is defined as 'the duration of time from initiation to discontinuation of therapy' [4].

Compliance to and persistence on medication may be monitored through different methods, direct and indirect, each one with particular advantages and disadvantages [2,5]. No method is considered the gold standard. Patient self-reports are simple, inexpensive and the most useful method in the clinical setting [6,7], although higher rates of adherence/persistence have been reported when patient self-reports are used for evaluation [5].

Adherence rates are typically higher among patients with acute conditions as compared with those patients with chronic conditions. Persistence of adherence among patients with chronic conditions is disappointedly low and drops dramatically after the first 6 months of therapy [8-10]. Efforts had been made to develop a questionnaire to investigate patient compliance and persistence with antirheumatic drugs [11-13], but few studies have examined the topic in chronic inflammatory rheumatic conditions – the majority of these studies focused on RA treatment. In those studies, different definitions of adherence and persistence have been used and different populations and medications evaluated, thus limiting any conclusions. The current literature, however, suggests that nonadherence to and nonpersistence on DMARD therapy is a substantial problem, ranging in occurrence from 20% to 70% [10,14-23].

Many factors have been related to patient's medication behavior in RA patients, including younger age [16,21,23], male sex [20,21], belonging to an ethnic minority [17], lower education [17], side effects [17], availability of financial resources and social support [18,23], medication-taking behavior and beliefs [19], increased disability [20], better perceived health status at the beginning [10], poor quality of contact with health professionals [20], poor personal knowledge about the disease and its treatment [20], comorbidity [23] and the class of DMARDs [10,15,22].

Compliance and persistence with prescribed medication regimens (and placebo regimens) predict better outcomes. Collecting adherence and persistence data from patients is considered an essential part of clinical trials.

By contrast, poor compliance and nonpersistence with medication contribute to substantial worsening of disease and death, and increase healthcare costs [2].

We report our experience with persistence on DMARDs and corticosteroids evaluated through patient–physician structured interviews, during 2 years of follow-up of an early RA cohort of patients. The aims of the study were to determine persistence on DMARDs and corticosteroids over 13 consecutive visits each 2 months apart in a cohort of early RA patients, to investigate the relationship between persistence on therapy and disease activity, to investigate the relationship between persistence on therapy and both disability and structural damage, and to identify whether any baseline factors are associated with nonpersistence on medication.

## Materials and methods

### Setting and study population

The Instituto Nacional de Ciencias Médicas y Nutrición Salvador-Zubirán is a referral centre for Rheumatic Diseases in México City. In February 2004, an early arthritis clinic was established. Patients with disease duration of less than 1 year and nonspecific rheumatic diagnosis at initial evaluation but with RA attended the clinic. As part of the standard care provided, patients were evaluated at baseline and every 2 months by the same rheumatologist.

For the present report, we included data for all patients who attended the clinic for at least 24 months (13 consecutive evaluations scheduled) up to March 2008: 70 patients completed 2-year follow-up; additionally, three patients were lost to follow-up before visit 3, one before visit 8 and another patient before visit 13. Their data available up to the last observation were also included in the analysis. As persistence on therapy was evaluated from the second visit we excluded from the analysis four additional patients who were lost to follow-up after baseline evaluation.

### Clinical evaluations

Standard baseline and follow-up evaluations included, at baseline, a complete demographic and medical history obtained by face-to-face interview, a rheumatic evaluation that assessed 66 swollen joint counts and 68 tender joint counts, and a physician global assessment of disease activity on a 100 mm visual analogue scale. Before the medical evaluation, a Hispanic version of the health assessment questionnaire (HAQ) [24] and two 100 mm visual analogue scales, one for pain and one for overall disease activity, were completed by patients. Laboratory investigations included, at minimum, determination of rheumatoid factor and C-reactive protein serum levels (both by nephelometry), a second-generation ELISA for antibodies to cyclic citrullinated peptides and the erythrocyte sedimentation rate by the Westergren method. Disease activity scores were calculated using the 28-joint disease activity score (DAS28) [25].

For the follow-up evaluations, the HAQ, the 100 mm visual analogue scale for pain, the 100 mm visual analogue scale for overall disease activity and the physician global assessment of disease activity on a 100 mm visual analogue scale were completed by patients and the physician, who additionally performed 66 swollen joint counts and 68 tender joint counts and scored the DAS28. The erythrocyte sedimentation rate and C-reactive protein measurements were determined.

Information concerning comorbidities was established by record review, based on physician diagnosis; in addition, when a patient was given treatment for a specific diagnosis not recorded on the charts (for instance, antihypertensive therapy), the corresponding section was updated. Counted comorbidities were: arterial pulmonary hypertension, arterial systemic hypertension, asthma, cardiovascular disease, diabetes mellitus, end-stage renal disease, epilepsy, glaucoma, heart block, hepatic cirrhosis, hepatitis virus B infection, hepatitis virus C infection, hydrocephaly, hyperlipidemia, major depression, obesity, osteoarthritis, osteoporosis, psoriasis, pulmonary fibrosis, thyroid disorder and vitiligo.

At every visit the same rheumatologist performed a predefined interview regarding prescriptions. Patients were directed to refer to the name(s), dose(s) and schedule(s) of the drug(s) (DMARDs, corticosteroids and other) they had been taking since the last visit (2 months apart), initially spontaneously and if necessary directly. Patients were then asked about any missing/incorrect medication, dose and/or schedule since the previous visit; emphasis was placed on DMARDs and corticosteroids. The number of days of missing medication was also recorded. The rheumatologist compared the last prescription and actual treatment; if inconsistencies were found, they were solved.

Data were collected in standardized formats.

### Radiography

Digitized images of radiographs of the hands and feet (postero-anterior and oblique views) were scheduled at baseline, at 1-year and at 2-year follow-up, and were read in chronological order by a radiologist and a rheumatologist. RA was classified as erosive disease (at least one cortical bone defect) or nonerosive disease, once consensus was reached.

### Treatment

Treatment was recorded in standardized formats, including the use of corticosteroids (yes/no), the use of DMARDs (yes/no), the number and name(s) of DMARDs/patient and the treatment prescribed for comorbidities. Records included *previous treatment *(during the month prior to baseline evaluation) prescribed by physicians who referred patients to the clinic, *baseline treatment *prescribed by the rheumatologist in charge of the clinic at first evaluation, and *treatment prescribed at each follow-up visit*.

At baseline and consecutive visits, adverse events were recorded as part of the standard care provided. Treatment modifications because of adverse events were not considered as nonadherence/nonpersistence when indicated by a physician.

### Definitions

*Nonpersistence with medication *was defined as the duration of time from initiation to discontinuation of DMARDs and/or corticosteroids of at least 7 consecutive days. Regarding methotrexate, at least one weekly missing dose was considered to meet the nonpersistence definition. Nonpersistence with therapy was evaluated from the second visit and was defined by an independent observer according to the information recorded on the charts.

*Sustained remission* was defined at last visit, as three or more consecutive visits each 2 months apart with a DAS28 below 2.6.

### Ethics

Patients agreed to enter the Early Arthritis Clinic and approved clinical and radiological assessments. Treatment was prescribed by the rheumatologist in charged of the Early Arthritis Clinic according to patient and disease characteristics. The study was approved by the Institution Review Board. Written informed consent was obtained from all participating patients.

### Statistics

By definition, persistence is reported as a continuous variable in terms of the number of days for which therapy was available, although it may also be reported as a dichotomous variable measured at the end of a predefined time period [4]. Accordingly, data are presented as the number (%) of patients being persistent (or nonpersistent) at every consecutive visit.

Descriptive statistics, Student's *t *test and the chi-squared test were used as appropriate. Multivariate logistic regression analyses were conducted. To summarize the serial clinical, serological, treatment and comorbidity measurements, areas under the curve were calculated by the trapezoid method and are presented standardized by the length of the study [26]. To identify baseline predictors of nonpersistence with medication, different models were constructed. At first, variables that were significant at *P *< 0.20 on the univariate analysis were entered into the multivariate model (age, erythrocyte sedimentation rate and C-reactive protein). We also included in the final model the baseline variables reported in the literature to be associated with poor adherence/persistence: male sex, lower education, socioeconomic status, disability (baseline HAQ), perceived health status (patient 100 mm visual analogue scale for overall disease activity) and comorbidity [10,14-20,22,23] – although all of them showed *P *> 0.20 in the univariate analysis. At the beginning, saturated models were tested and the less significant variables were excluded. Finally, the parsimonious models are reported. Two-tailed *P *≤ 0.05 was considered significant. Analyses were performed using the SPSS/PC program (version 12.0; SPSS Inc., Chicago, IL, USA).

## Results

### Persistence on medication survival function

By March 2008 data from 75 patients had been analyzed and 935 notes reviewed. Of these, 860 notes were evaluated (75 baseline notes excluded) and 71 (8.3%) notes with therapy discontinuation were identified; most of those notes (80.3%) recorded that patients were nonpersistent with one DMARD – 29 of them (40.8%) with methotrexate, 23 patients (32.4%) with chloroquine, 15 patients (21.1%) with sulphasalazine, 11 patients (15.5%) with penicillamine, eight patients (11.3%) with leflunomide, six patients (8.5%) with minocicline and one patient (1.4%) with prednisone.

Forty-three patients (57.3%) were nonpersistent on at least one evaluation and their (mean ± standard deviation (SD)) days of therapy discontinuation were 41.7 ± 25.9. There were incident nonpersistent patients at each consecutive visit, their numbers ranging from one patient at visit 7 to seven patients at visit 4. Among nonpersistent patients, 24 (55.8%) were nonpersistent at one evaluation and their (mean ± SD) days of therapy discontinuation were 28.9 ± 16.6; 11 patients (25.6%) were nonpersistent at two evaluations and accumulated (mean ± SD) 47.6 ± 21.5 days of therapy discontinuation; seven patients (16.3%) were nonpersistent at three evaluations and had (mean ± SD) 65 ± 22.4 days of therapy discontinuation; and finally, one patient (2.3%) was nonpersistent at four visits and his global period of therapy discontinuation was 119 days.

**Figure 1:**
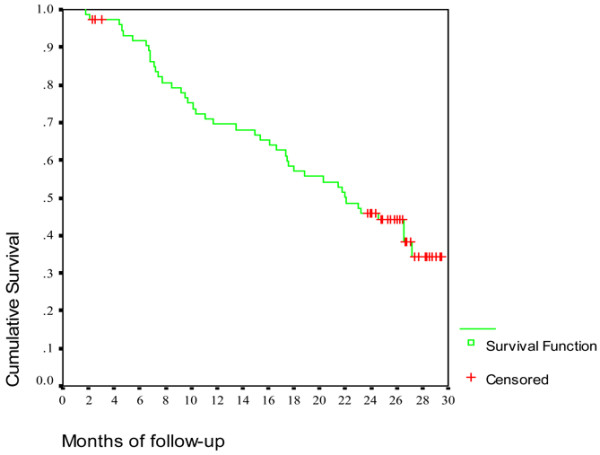
Cumulative risk of being persistent on therapy over 2 years of follow-up. Persistence decreased from 98% at visit 2 (2.15 ± 0.44 months of follow-up) to 34% at visit 13 (24.6 ± 6.17 months of follow-up).

Figure 1 shows the cumulative risk of being persistent on therapy, which decreased from 98% at visit 2 (2.15 ± 0.44 months of follow-up) to 34% at visit 13 (24.6 ± 6.17 months of follow-up).

### Baseline characteristics of the population studied

As shown in Table 1, baseline demographic and disease characteristics, comorbidities, baseline treatment and previous treatment (during the month previous to the baseline evaluation at the clinic) were the same in persistent patients and nonpersistent patients but the persistent patients were younger. Forty-eight percent of the patients from both groups had baseline positive antinuclear antibodies; fine and gross speckle were the most frequently reported patterns (68%). The dose range of current use of corticosteroids at baseline evaluation was between 5 and 15 mg/day (equivalent to oral prednisone).

**Table 1:** Baseline demographic and disease characteristics, treatment and comorbidities between persistent and nonpersistent patients

Variable	Persistent-patients (n = 32)	Nonpersistent patients (n = 43)	*P *value
Socio-demographic?			
Female sex (n (%))	27 (84.4%)	36 (83.7%)	1
Age at baseline evaluation (years)	36.1 ± 12.6	42.5 ± 13.7	0.04
Years of education	10.2 ± 3.6	10.1 ± 3.8	0.90
Low socioeconomic status (n (%))	27 (84.4%)	38 (88.8%)	0.87
Single (n (%))	17 (53%)	18 (42%)	0.36
Disease characteristics			
Number of American College of Rheumatology criteria	5.3 ± 0.7	5 ± 1.2	0.23
Time since first symptom (months)	5.5 ± 2.9	5.2 ± 2.7	0.55
Disease activity score (28 joints)	6.3 ± 1.2	6.2 ± 1.3	0.65
Health assessment questionnaire (0 to 3)	1.5 ± 0.8	1.5 ± 0.9	0.91
Physician global assessment of disease activity visual analogue scale (0 to 100)	49.1 ± 23	43.8 ± 20.9	0.30
Patient pain visual analogue scale (0 to 100)	60.4 ± 24.8	58.6 ± 26	0.76
Patient overall disease visual analogue scale (0 to 100)	62.1 ± 28	61.1 ± 26.4	0.87
Erythrocyte sedimentation rate (mm/hour)	36.6 ± 26.8	29.8 ± 18.1	0.19
C-reactive protein (mg/dl)	3.4 ± 5.1	2.2 ± 2.9	0.17
Patients with rheumatoid factor (n (%))	23 (71.9%)	30 (69.8%)	1
Patients with antibodies to cyclic citrullinated peptides (n (%))	22 (68.8%)	29 (69%)^a^	1
Patients with comorbidity (n (%))	12 (38%)	15 (35%)	1
Baseline treatment at the clinic			
Corticosteroid use (n (%))	9 (28.1%)	11 (25.6%)	1
Number of DMARDs/patient	2 ± 0.7	1.8 ± 0.8	0.36
Number of drugs for comorbid conditions/patient	1.75 ± 1.3	1.4 ± 0.8	0.40
Number of total drugs/patient	3.6 ± 1.3	3.4 ± 1.2	0.56
Previous treatment			
Corticosteroid use (n (%))	4 (12.5%)	9 (29.9%)	0.38
DMARD use (n (%))	11 (34.4%)	12 (27.9%)	0.61
Number of DMARDs/patient (among users)	1.5 ± 0.5	1.4 ± 0.5	0.86

Data presented as the mean ± standard deviation unless otherwise indicated. DMARD, disease-modifying antirheumatic drug. ^a^One missing data.

### Relationship between persistence on medication and disease activity

Standardized areas under the curve (mean ± SD) for clinical and serological (erythrocyte sedimentation rate and C-reactive protein) disease activities were similar between both groups, as were areas under the curve for the number of comorbidities/patient and the number of drugs/patient (Table 2).

**Table 2:** Standardized AUCs for clinical, serological, comorbidity and treatment serial measurements of persistent and nonpersistent patients

AUC for serial assessments	Persistent-patients (n = 32)	Nonpersistent patients (n = 43)	*P *value
Clinical			
Disease activity score (28 joints)	2.83 ± 1.45	3.04 ± 0.89	0.45
66 swollen joint counts	3.65 ± 5.06	3.99 ± 2.86	0.72
68 tender joint counts	4.02 ± 5.16	3.94 ± 2.74	0.94
Physician global assessment of disease activity visual analogue scale	11.72 ± 16.46	12.24 ± 7.60	0.87
Patient pain visual analogue scale	11.79 ± 11.42	12.77 ± 7.24	0.65
Patient overall disease visual analogue scale	11.73 ± 12.3	13.19 ± 7.26	0.52
Health assessment questionnaire	0.23 ± 0.36	0.36 ± 0.32	0.23
Serological			
Erythrocyte sedimentation rate	16.99 ± 11.97	15.99 ± 9.67	0.69
C-reactive protein	1.18 ± 1.52	0.73 ± 0.69	0.13
Number of comorbidities/patient	1.33 ± 2.4	1.1 ± 1.48	0.62
Treatment			
Number of DMARDs/patient	2.31 ± 0.71	2.47 ± 0.71	0.35
Number of drugs for comorbidity/patient	1.81 ± 1.03	1.83 ± 0.88	0.90
Number of total drugs/patient	4.38 ± 1.13	4.61 ± 1.17	0.39

Data presented as the mean ± standard deviation. AUC, area under the curve; DMARD, disease-modifying antirheumatic drug.

**Figure 2:**
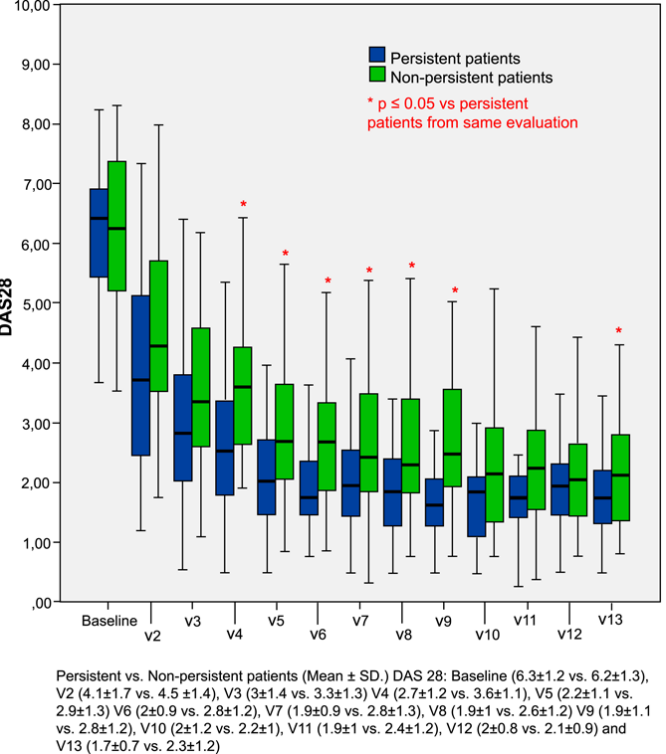
Comparison of consecutive 28-joint disease activity scores between persistent patients and nonpersistent patients. Consecutive 28-joint disease activity score (DAS28) for persistent patients (blue bars) and nonpersistent patients (green bars). Thick line in middle of bar, mean DAS28 value; top and bottom of bar, upper and lower quartiles, respectively; top and bottom lines, maximum and minimum values, respectively. *x *axis, consecutive visits (V); *y *axis, DAS28 values. *Differences with *P *≤ 0.05. SD, standard deviation.

Persistent patients had similar (mean ± SD) DAS28 at baseline and at visits 2 and 3 to nonpersistent patients. After 6 months of follow-up, persistent patients had lower (mean ± SD) consecutive DAS28 than nonpersistent patients and the differences were statistically significant at visits 4 to 9 and at visit 13 (*P *≤ 0.03), as shown in Figure 2. In addition, persistent patients had greater improvements in disease activity (measured by the difference between the DAS28 at the corresponding visit and the baseline DAS28) at every consecutive visit than nonpersistent patients; differences were statistically significant at visit 4 (3.5 ± 1.5 versus 2.6 ± 1.5, *P *= 0.009), at visit 6 (4.2 ± 1.5 versus 3.4 ± 1.6, *P *= 0.04), at visit 7 (4.2 ± 1.5 versus 3.4 ± 1.6, *P *= 0.04) and at visit 9 (4.3 ± 1.5 versus 3.4 ± 1.5, *P *= 0.01).

Sustained remission definition (at least three consecutive visits with DAS28 < 2.6) required at least 6 months of follow-up. Seventy-two patients met the follow-up required: 70 patients completed 13 consecutive visits, one patient underwent seven consecutive visits and another patient completed 12 consecutive visits. More persistent patients had sustained remission at last follow-up than nonpersistent patients (24 (82.8%) versus 20 (46.5%) patients, *P *= 0.003). Among patients who achieved sustained remission, persistent patients had shorter follow-up to outcome than nonpersistent patients (7.7 ± 4.5 versus 13.6 ± 5.7 months, *P *= 0.001).

### Relationship between persistence on medication and disability

**Figure 3:**
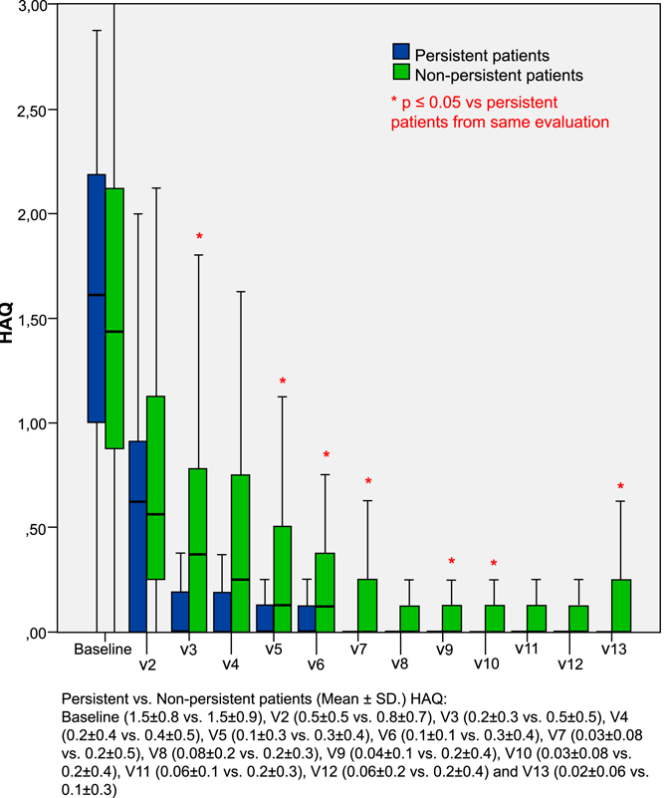
Comparison of consecutive health assessment questionnaire scores between persistent patients and nonpersistent patients. Consecutive health assessment questionnaire (HAQ) scores for persistent patients (blue bars) and nonpersistent patients (green bars). Thick line in middle of bar, mean value; top and bottom of bar, upper and lower quartiles, respectively; top and bottom lines, maximum and minimum values, respectively. *x *axis, consecutive visits (V); *y *axis, HAQ values. *Differences with *P *≤ 0.05. SD, standard deviation.

Both groups of patients had similar standardized areas under the curve for the HAQ (Table 2). Persistent patients had similar baseline (mean ± SD) HAQ scores to nonpersistent patients. At each follow-up visit, persistent patients had lower (mean ± SD) HAQ scores than nonpersistent patients and the differences were statistically significant at visit 3, at visits 5 to 7, and at visits 9, 10 and 13, as shown in Figure 3. Finally, persistent patients had shorter follow-up to the first visit with HAQ score ≤ 0.20 than nonpersistent patients (mean ± SD, 5.2 ± 4 versus 8.2 ± 5.7 months, *P *= 0.02).

### Relationship between persistence on medication and erosive disease

We compared persistence on medication between patients who did and who did not develop erosive disease according to radiography. One nonpersistent patient had erosive disease at baseline and was discarded from the analysis. Five additional patients did not complete the 2-year follow-up and did not have corresponding X-ray scans – their data were also excluded. Finally, 69 patients had X-ray scans performed at 2 years of follow-up: 28 were persistent and 41 were not. More nonpersistent patients developed erosive disease than persistent patients but the difference did not reach statistical significance (5 (17.9%) versus 11 (26.8%), *P *= 0.56).

### Baseline factors associated with nonpersistence on medication

In the multivariate model, among the 43 nonpersistent patients, older age was the only variable to be consistently associated with nonpersistence on therapy (odds ratio = 1.1, 95% confidence interval = 1.007 to 1.103, *P *= 0.02) and was still associated after controlling for early (during the first 12 months of follow-up) nonpersistence versus late (after 12 months of follow-up) nonpersistence. Similar results were obtained when the model was applied in the 24 patients who were nonpersistent at only one evaluation during their 2-year follow-up (older age, odds ratio = 1.04, 95% confidence interval = 1.002 to 1.087, *P *= 0.04).

## Discussion

The terminology, definitions and methods to determine adherence and persistence differ greatly in the published literature [27]. A need for improvement in the quality and consistency of medication compliance and persistence research is mandatory. In 2006 the International Society of Pharmaeconomics and Outcomes Research published a consensus document that was intended to improve the consistency and quality of analysis regarding this topic and to understand the impact of compliance and adherence on health outcomes [28]. Two years later, Cramer and colleagues provided specific definitions for compliance and persistence, and encouraged their adoption by health outcome researchers [4]. In the present study we have adopted those definitions and analyzed persistence on DMARDs and corticosteroids in early RA patients. Nonetheless, poor adherence may be related to therapy discontinuation (that is, nonpersistence according to the recent proposal) and most of the published studies do not include persistence as a separate construct from compliance, which makes difficult a comprehensive discussion on the topic.

The present study highlights the impact of therapy persistence on different RA outcomes. After 6 months of follow-up, persistent patients showed lower scores and greater improvements in disease activity and disability and had more frequent and earlier sustained remission than nonpersistent patients. It may be argued that it is not clear whether better outcomes were a cause of or an effect of persistence on therapy. Nonetheless, at initial evaluations both groups of patients had similar clinical status, demography (except age), comorbidities, and disease characteristics. Treatment at baseline and during follow-up did not differ between them, suggesting that greater clinical improvement in persistent patients was probably related to persistence on therapy. To the best of our knowledge there is only one study that has evaluated compliance and RA outcomes. Viller and colleagues showed more frequent improvement in disability (no relationship with disease activity was investigated) in consistently compliant European RA patients over 3 years of follow-up than in those who changed behavior [21]. They included 556 patients with < 5 years of disease duration, and compliance with drug dosages and dosing times was assessed yearly using a questionnaire. We also found that more nonpersistent patients developed erosive disease than persistent patients, although the difference was not statistically significant – we were probably limited by the number of patients with erosive disease. Interestingly, most nonpersistent patients (56%) omitted treatment once during follow-up; among them, the number of therapy-discontinuation days was low (4% of the indicated days of therapy) but impacted negatively on the disease prognosis.

Among potential candidates, older age was the only predictor to be associated with nonpersistence on DMARD and corticosteroid therapy. Kristensen and colleagues identified high age (in addition to low C-reactive protein serum level, elevated HAQ score, and higher previous number of DMARDs) as a predictor of premature treatment termination (which may be considered a synonym for nonpersistence) with etarnecept and infliximab in 1,161 patients with active RA [29]. By contrast, Viller and colleagues found older age (in addition to female sex, decreased disability, very satisfactory contacts with health professionals, and more personal knowledge about the disease and its treatment) significantly associated with good compliance in their early RA cohort [20]. Self-reporting of *current *medication use with DMARDs (methotrexate, sulphasalazine, and corticosteroids) has been considered to have good to excellent agreement with information obtained from the medical charts [30]. In our study, the time between two visits was 2 months so the treatment recorded could be considered current treatment; meanwhile, compliance with drugs was assessed annually in the study of Viller and colleagues. Tuncay and colleagues performed three assessments for drug compliance over 1 year of follow-up in 100 RA patients [16]. Consistently compliant patients were older than consistently noncompliant patients, although no regression analysis was performed. In both studies, patients had longer disease duration than patients from our study and the concept of medication persistence as a different construct from medication adherence was not defined. Finally, Curkendall and colleagues showed that persistence on (and adherence with) anti-TNFα was better among older patients, in an inception cohort of anti-TNFα-naïve-RA patients [23]; worse persistence was also associated with greater out-of-pocket costs, with higher Charlson's comorbidity score and with previous prescription for a narcotic analgesic. Adherence and persistence were measured using claims data on a particular RA population of unknown disease duration, which may have accounted for the different result.

Persistence on therapy decreased in our cohort of early RA patients up to 34% at 2 years of follow-up. Curkendall and colleagues showed that persistence on (and adherence with) anti-TNFα decreased over 1 year of follow-up in their inception cohort of RA patients, up to 32% in those patients with out-of-pockets costs above $50/week [23]. Similar results were found regarding a decline of compliance by de Klerk and colleagues in 127 outpatients, 81 of whom had RA [10]; by Viller and colleagues, who identified 35.7% of consistently compliant patients after three years of follow-up [20]; and by Tunkay and colleagues, who found that 30.2% of 100 RA patients were consistently compliant over 1 year of follow-up [16]. Two other studies performed in 66 patients and 108 RA patients, respectively, showed higher rates of compliance and persistence with therapy, ranging from 61% to 73% [15,31]. Numerous randomized clinical trials performed in RA patients had also assessed drug continuation (persistence) over different follow-up periods [32-38], although the probability of discontinuation for reasons other than adverse events and lack of efficacy was very low.

The present study has several limitations. We did not use a well-validated questionnaire scale to assess compliance/persistence [11,13,39]. We assessed persistence through the simplest and most used way in clinical practice, which is to ask the patient whether he/she is still taking the medication as prescribed [7,40-43]. We used a partially arbitrary and recently proposed definition to define nonpersistence. We choose a lag time of 1 week, as 89% of our patients were taken methotrexate and the drug is indicated weekly. We analyzed neither the whole spectrum of nonadherence/nonpersistence nor its repercussion on disease activity and damage – clinical outcomes of treatment are affected not only by how long patients take their medication but also by how well; accordingly, adherence should have been defined and measured to characterize medication-taking behavior comprehensively. In terms of guaranteeing a better prognosis, how to define a reasonable adherence/persistence is still unknown. Finally, the outcome is not the result of compliance and persistence alone but can be influenced by many other factors [44]. In that sense, the present study was carried out in an inception cohort of early RA patients, with particular sociodemographic characteristics, ethnicity, treatment and health system, and our results may not be generalized to RA populations with different characteristics [45].

In 2006, the American College of Rheumatology endorsed a starter set of quality indicators for several rheumatic diseases, among them RA [46,47]. Two measures pertinent to treatment were included and strategies proposed to be used when there is increased disease activity or damage progression over a 6-month period. The recommendations add potential adverse events, increase costs, and are based on the assumption that patients are fully (or partially) compliant with therapy and persistent on medication. The present study reinforces the necessity to consistently investigate compliance and persistence with treatment before regimens are modified. Potential benefits are better prognosis, cost reductions and patient safety.

## Conclusion

Nonpersistence on DMARDs was frequent and progressive over the first 2 years of follow-up in a cohort of early RA patients, a time frame that has been proposed as crucial for disease control/remission. Persistent patients were younger at baseline evaluation and had lower disease activity and disability during their follow-up than nonpersistent patients. Persistent patients reached sustained remission more frequently and earlier than nonpersistent patients. Persistence and adherence with medication should routinely be evaluated during RA follow-ups, especially at the beginning of the disease when adequate treatment has a major impact on disease outcomes.

## Abbreviations

DAS28: 28-joint disease activity score; DMARD: disease modifying anti-rheumatic drug; HAQ: health assessment questionnaire; MP: medication persistence; RA: rheumatoid arthritis.

## Competing interests

The authors declare that they have no competing interests.

## Authors' contributions

VP-R performed conception and design, data acquisition, analysis and interpretation of the data, drafting and critical review. IC-Y performed conception and design, data acquisition, and analysis and interpretation of the data. ARV-R performed analysis and interpretation of the data. JC performed data acquisition, and analysis and interpretation of the data. MR-G performed data acquisition, analysis and interpretation of the data and critical review.
